# Improving Documentation and Follow-Up of Elevated Blood Pressure in a Family Clinic: A Quality Improvement Project

**DOI:** 10.7759/cureus.89711

**Published:** 2025-08-09

**Authors:** Mayank Korpal, Neil Jaddou

**Affiliations:** 1 Medicine, Government Medical College, Amritsar, IND; 2 Family Medicine, Corewell Beaumont, Henry Ford Hospital, Troy, Royal Oak, USA

**Keywords:** blood pressure documentation, electronic health record, family medicine clinic, follow-up planning, hypertension management, outpatient care, plan do study act, quality improvement, team huddles, verbal counseling

## Abstract

Introduction

Hypertension is a common and clinically significant condition frequently encountered in primary care. However, challenges such as poor documentation and inconsistent follow-up planning in many outpatient settings can result in suboptimal outcomes, increasing the risk of missed care opportunities. This quality improvement project aimed at improving documentation and follow-up planning for patients with elevated blood pressure (BP) (>140/90 mmHg) at a family clinic in Michigan.

Objective

This quality improvement project is aimed at improving documentation and follow-up planning for elevated BP readings in adult patients seen during outpatient visits from 33% to 70% over a three-week period in a family medicine clinic.

Methods

The project was conducted at an outpatient family medicine clinic over a three-week period from June 9 to June 27. Adult patients aged 18 years and older with elevated BP were included, and a total of 60 patient charts were reviewed during the intervention period. The intervention consisted of a daily review of patient charts to identify elevated BP, ensuring that follow-up plans such as home BP monitoring, repeat BP checks, and lifestyle modification advice were documented in the electronic health record (EHR). Patients with elevated readings also received verbal counseling, and brief end-of-day team huddles were conducted to review documentation, and early in the cycle, brief staff education sessions were held to review the documentation standards. Data were collected through both EHR review and manual chart audits. A Plan-Do-Study-Act (PDSA) cycle was used to implement and evaluate the intervention.

Results

At baseline, only 33% (20 out of 60) of the patients with elevated BP had appropriate documentation and follow-up plan in the EHR. Following the three-week intervention, this increased to 80% (48 out of 60), surpassing the initial target of 70%. The documentation improvement was achieved using the iterative PDSA cycle approach, with adjustments made weekly to reinforce chart review, counselling, and end-of-day team huddles.

Conclusion

This quality improvement cycle led to a significant improvement in the documentation and follow-up plans, highlighting its importance in better management of hypertension. The intervention has been sustained in the daily clinic practice, with minor adjustments made to support long-term sustainability. This may also serve as a model for similar clinic-based quality improvement efforts.

## Introduction

Hypertension is one of the major contributors to cardiovascular morbidity and mortality. According to the Centers for Disease Control and Prevention (CDC), nearly half of U.S. adults (119.9 million) have hypertension, yet only one in four have it adequately controlled [1]. Despite growing awareness, only around 20% of hypertensive individuals achieve blood pressure (BP) control under 130/80 mmHg [2]. Poorly controlled hypertension contributes significantly to cardiovascular events, including myocardial infarction, stroke, heart failure, and chronic kidney disease [3]. Throughout this study, the term “elevated BP” is used in line with Eighth Joint National Committee (JNC 8) guidelines (≥140/90 mmHg), although we acknowledge that “hypertension” is often used interchangeably in the literature, particularly per the American College of Cardiology/American Heart Association (ACC/AHA) 2017 definitions, which define hypertension as ≥130/80 mmHg.

There is a wide acknowledgement regarding the clinical implications of proper documentation and follow-up for elevated BP. Multiple studies support the role of systematic follow-up in improving control of hypertension [4]. Initiatives such as the Centers for Medicare and Medicaid Services (CMS) Quality Measure 317 underscore the necessity of formal documentation and follow-up for elevated BP as a quality benchmark [5].

In the outpatient setting, incidental elevated BP readings are frequently overlooked, especially when patients visit for unrelated issues. Our urban family medicine clinic, located in Troy and Sterling Heights, Michigan, identified this issue through routine audits and subsequently initiated a quality improvement (QI) project.

Similar QI models have demonstrated success using approaches such as telephone counseling, clinical reminders, and care team interventions [6-8]. This intervention was informed by similar QI models, such as the Million Hearts campaign, the SPRINT trial, and EHR-based decision support strategies, which have shown improved documentation and follow-up of elevated BP in outpatient settings. These interventions not only improve documentation rates but also enhance clinical outcomes [9,10]. Therefore, a simple, low-cost, team-based QI intervention was designed to improve the consistency of documenting elevated BP and ensure appropriate follow-up.

## Materials and methods

Setting

This QI project was conducted at Somerset Family Medicine Clinic, which includes two outpatient locations situated in Troy and Sterling Heights, Michigan. Both clinics serve a diverse urban population and offer comprehensive primary care services to the patients. The setting provided a practical environment for implementing system-level changes, supported by a multidisciplinary care team comprising attending physicians, nurses, and administrative staff.

Population

The intervention targeted adult patients aged 18 years and older who had elevated BP readings documented during their visits. Elevated BP was defined in accordance with the JNC 8 guidelines [6] as systolic BP ≥140 mmHg or diastolic BP ≥90 mmHg. Over the course of the project, a total of 60 patient charts were reviewed and included in the QI cycle. These charts were selected based on patient encounters during the project period in which elevated BP was recorded, regardless of the primary reason for the visit.

Design

A single Plan-Do-Study-Act (PDSA) cycle was implemented over a three-week period from June 9 to June 27, 2025. This cycle was designed to test and refine a workflow intervention aimed at improving documentation and follow-up planning for elevated BP. Since the initiative focused on internal QI and did not involve experimental procedures or direct patient contact for research purposes, it did not meet the criteria for human subject research. Therefore, Institutional Review Board (IRB) approval was not required. The short, focused timeline was chosen because of rapid-cycle evaluation and to minimize disruption to clinical services. Although only one formal PDSA cycle was conducted over the three-week period, iterative refinements such as updated staff reminders, evolving team huddle content, and real-time feedback were embedded into the cycle to enhance responsiveness.

Intervention steps

The intervention involved several coordinated activities that were integrated into daily clinical operations. Patient charts were reviewed each day to identify encounters where elevated BP readings had been recorded. Then, a structured health plan was recorded in the electronic health record (EHR), consisting of recommendations such as repeat in-clinic BP measurements, home BP monitoring, and lifestyle modification counseling. Additionally, end-of-day team huddles were conducted to review selected charts for completeness. These meetings typically lasted about 10 minutes and focused on reinforcing best practices, identifying documentation gaps, and fostering a sense of shared accountability. Early in the cycle, brief staff education sessions were held to review the documentation standards outlined by the CMS, particularly the CMS22v13 electronic Clinical Quality Measure (eCQM), which emphasizes the importance of documenting follow-up care for patients with elevated BP. To maintain adherence, daily team huddles were used not only for review but also for reinforcing compliance. Brief reminders were sent via EHR, and informal huddle attendance was tracked to ensure consistency.

Data collection

Data collection was performed using a combination of EHR-based analytic tools and manual chart audits. The documentation rate, which served as the primary outcome measure, was defined as the proportion of charts in which both elevated BP was recognized and an appropriate follow-up plan was explicitly documented. Baseline documentation rates were established before the intervention began, and final rates were assessed at the end of the three-week intervention period. Data reviewers adhered to a standardized review protocol to ensure consistency and reliability in data extraction and outcome classification.

Statistical consideration

Due to the small sample size of 60 patient encounters, the analysis was limited to descriptive statistics. Pre- and post-intervention documentation rates were calculated and reported as simple percentages. No inferential statistical testing was conducted, as the primary aim of this project was to assess feasibility, test workflow changes, and evaluate short-term process improvement. Nonetheless, the difference observed between baseline and post-intervention documentation rates was considered meaningful in the context of QI, particularly given the limited duration and resources required for implementation.

Ethical consideration

This project was undertaken as a QI project that did not meet the criteria for human subject research. Therefore, Institutional Review Board (IRB) approval was not required. No patient identifiers were recorded or retained during chart review. Only de-identified, aggregate data were used for analysis.

## Results

A total of 60 patient charts with elevated BP readings (≥140/90 mmHg) were reviewed during the three-week intervention cycle. At the baseline, only 33% of these charts (20 out of 60) had appropriate documentation and a clear follow-up plan recorded in the EHR. This included documentation of repeat BP checks, home monitoring recommendations, or counseling on lifestyle modifications.

**Figure 1:**
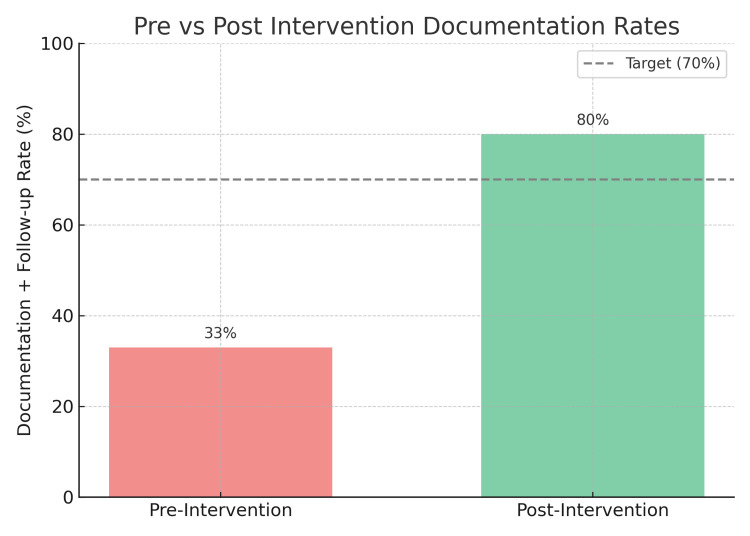
Pre- and post-intervention documentation and follow-up rate The bar chart shows the improvement in the rates of documentation and follow-up planning after the quality improvement intervention. The documentation rates increased from 33% at baseline to 80% post-intervention. The dotted line shows the initially decided target of 70%.

**Figure 2:**
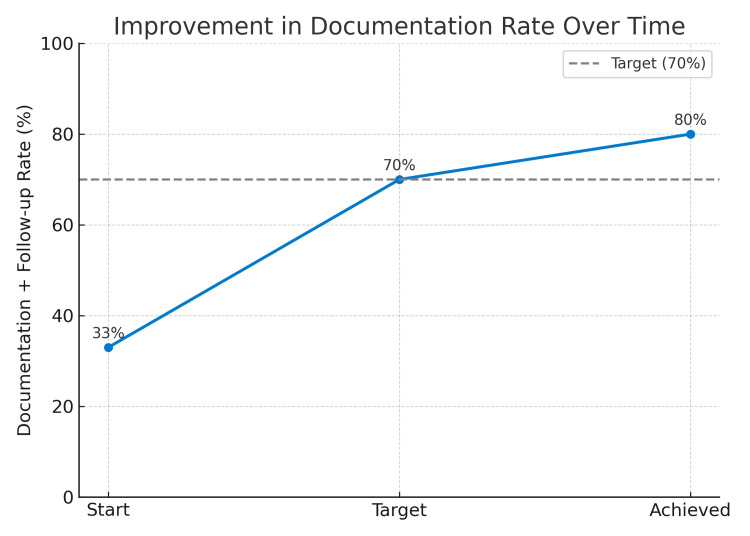
Progress in the documentation and follow-up rates as compared to the target The line graph shows the improvement in the documentation and follow-up rate of patients with elevated BP from 33% to 80%, with the reference line indicating the initial target of 70%.

Following the implementation of a structured QI intervention, documentation rates improved to 80% (48 out of 60) by the end of the intervention cycle (Figure 1). This surpassed the original improvement target of 70% (Figure 2) and demonstrated the effectiveness of simple, low-cost strategies such as daily team huddles, verbal reminders, and standardized documentation practices.

The improvement was consistent and sustained throughout the project duration. No adverse patient events or complaints were reported during the intervention period. The project’s findings suggest that even short-term, resource-efficient interventions can significantly enhance documentation and care planning in a busy outpatient primary care setting.

## Discussion

This project demonstrated a substantial improvement in follow-up documentation for patients with elevated BP from 33% to 80% over three weeks, surpassing the initial 70% goal. These findings are aligned with previous QI initiatives that emphasize structured interventions, teamwork, and feedback loops to improve clinical care [7,9,11].

Evidence from the Million Hearts campaign and trials such as the SPRINT and ACCORD studies has reinforced the importance of early intervention and documentation in improving long-term cardiovascular outcomes [12,13]. Based on similar QI interventions and other published research, it can be seen that when care teams are engaged daily, especially using real-time feedback mechanisms such as huddles, provider behavior improves significantly [10,14].

One of the strengths of this project includes rapid and easy implementation, and, importantly, it was implemented within the existing workflow, which facilitated quick and easy adoption of the intervention. There was minimal disruption to clinical care, and the approach emphasized ownership and accountability without requiring extensive additional resources. This is especially relevant in the context of outpatient family medicine, where time and staff availability are often limited.

However, this project did have limitations, such as the short duration of the intervention that prevented long-term sustainability from being assessed. Furthermore, the outcome measures were process-based documentation and follow-up planning rather than clinical outcomes, such as actual BP control or cardiovascular event reduction. While improved documentation is an essential precursor to better management, further studies are needed to establish whether such process changes translate into improved clinical outcomes over time. Given the pre-post design of this QI initiative, it is possible that heightened awareness among staff during the intervention period may have influenced outcomes. This was partially mitigated by the use of a standardized data abstraction form and an objective binary outcome (presence/absence of documented follow-up). However, the structured nature of the intervention, consistency in documentation improvement, and integration into existing workflows suggest that the changes were likely related to the implemented strategies.

The implications of these findings extend beyond a single clinic. Numerous studies have reinforced that small, targeted QI projects, especially when they are contextually relevant and integrated into existing routines, can lead to substantial gains in performance metrics [15,16]. Given the simplicity, adaptability, and effectiveness of the intervention, this model may serve as a scalable framework for similar clinical environments. While only one formal PDSA cycle was conducted, several iterative adjustments were integrated within the three-week period, including reinforcement during daily huddles and targeted staff education that allowed for adaptive refinements without restarting the full cycle. Given that the primary objective of improving documentation and follow-up planning was surpassed within this time frame and that the intervention remained integrated into clinic routines thereafter, a single PDSA cycle was sufficient for this phase of the project. As health systems increasingly emphasize value-based care and population health metrics, improving foundational processes such as documentation and follow-up planning will be essential. The sustained success of this intervention suggests that with minimal investment, primary care clinics can measurably improve the quality of chronic disease management, enhance care coordination, and prepare for broader performance-based incentives tied to quality measures. To maintain gains, the clinic transitioned from daily to biweekly huddles and integrated documentation audits into the routine workflow. The model’s simplicity allows replication in other settings for chronic conditions such as diabetes, obesity, and hyperlipidemia, where documentation of care plans and monitoring parameters can similarly benefit from structured team-based review.

## Conclusions

This QI project led to a meaningful improvement in documentation and follow-up planning of patients with elevated BP in an outpatient family medicine clinic. This structured yet simple intervention proved to be a sustainable, effective, and important foundational step toward improved care coordination and management. Given the low resource requirement and adaptability of the interventions, it can be easily implemented in similar clinical environments. Future initiatives will focus on expanding and implementing this approach to other chronic conditions within the clinic to further enhance the patient outcome.
